# Correction to “Asbestosis Requiring Lung Transplantation in a Retired Hairdresser: An Occupational Exposure to Comb Through”

**DOI:** 10.1002/rcr2.70349

**Published:** 2025-09-22

R. Bu, L. Puttagunta, K. Halloran, et al., “Asbestosis Requiring Lung Transplantation in a Retired Hairdresser: An Occupational Exposure to Comb Through,” *Respirology Case Reports* 13, no. 7 (2025): e70283, https://doi.org/ 10.1002/rcr2.70283.

In paragraph 4 under the “Case Report” section, the text “25,100 fibres per cubic centimetre each year” was incorrect. This should have read: “25 to 100 fibres per ml‐years.”

In the sentence regarding asbestos exposure from talcum powder, the sentence “likely contributed to disease development” was incorrect. This should be corrected to “was an unlikely contributor to disease development.”

We apologize for these errors.

